# Is time-restricted eating a robust eating regimen during periods of disruptions in daily life? A qualitative study of perspectives of people with overweight during COVID-19

**DOI:** 10.1186/s12889-022-13856-9

**Published:** 2022-09-10

**Authors:** Natasja Bjerre, Lotte Holm, Jonas Salling Quist, Kristine Færch, Nana Folmann Hempler

**Affiliations:** 1 Health Promotion Research, Copenhagen University Hospital - Steno Diabetes Center Copenhagen, Borgmester Ib Juuls Vej 83, 2730 Herlev, Denmark; 2 Department of Food and Resource Economics, University of Copenhagen, Rolighedsvej 23, 1958 Frederiksberg, Denmark; 3 Clinical Research, Copenhagen University Hospital - Steno Diabetes Center Copenhagen, Borgmester Ib Juuls Vej 83, 2730 Herlev, Denmark; 4 Department of Biomedical Sciences, University of Copenhagen, Blegdamsvej 3B, 2200 Copenhagen, Denmark; 5 School of Psychology, University of Leeds, Woodhouse Lane, West Yorkshire, LS2 9JT Leeds, UK

**Keywords:** Time-restricted eating, Disruptions, Overweight, Weight loss, Daily life, Lifestyle, COVID-19 lockdown, Eating, Public health, Qualitative research

## Abstract

**Background:**

Time-restricted eating (TRE) has been suggested as a feasible dietary strategy in individuals with overweight. Disruptions in daily life e.g., severe illness can affect engagement in lifestyle interventions to obtain healthier body weight. This study examined if and how the engagement with TRE among people with overweight was affected by the Danish COVID-19 lockdowns as an example of disruptions in daily life.

**Methods:**

Fifteen participants with overweight enrolled in a TRE intervention, i.e. restricting all eating and drinking except water to the same daily ten-hour window, were interviewed about their experiences and engagement with TRE during COVID-19 lockdowns. Interviews were semi-structured and conducted by phone or face-to-face with safe social distancing. Data analysis was grounded in a reflexive thematic analysis approach.

**Results:**

Daily life rhythms were disrupted by lockdowns by preventing participants from performing ordinary daily activities such as going to work, socialising, eating out or exercising. For some, this challenged their TRE engagement, while most were able to undertake the TRE eating window but reported increased snacking and consumption of take-away food within their eating window. For all, exercise habits became unhealthier. The negative impact on TRE engagement primarily occurred during daytime, as social distancing made it easier to engage with TRE during evenings.

**Conclusions:**

This study showed that even people highly motivated to obtain healthier lifestyles practices struggled to maintain engagement with healthy behaviours, whereas sticking to the TRE window was manageable during COVID-19. TRE as a weight loss strategy was challenged which calls for more attention to supporting people in daily life to obtain healthier practices, also in case of periods of other disruptions such as divorce, serious illness etc.

## Background

The implementation of intermittent fasting regimens such as time-restricted eating (TRE) during periods of disruptions in daily life have been highlighted as a potential solution to reduce unhealthy eating and increase healthier habits [1]. TRE has been conceptualised as a simple strategy for losing weight and improving cardiometabolic health [2–4] as it allows people their usual intake of food and beverages, but limits this to a shorter daily interval (e.g. 10 hours) without any other restrictions [2, 5–7]. Human pilot intervention studies have reported moderate weight loss and improvements in other cardiometabolic risk factors including blood pressure, glucose tolerance and blood lipids in response to TRE interventions in individuals with overweight and high risk of type 2 diabetes [4, 8, 9]. However, there is a lack of randomised controlled trials and previous studies have not included comprehensive qualitative assessment of TRE feasibility in everyday life. Studies have found TRE to be appealing to perform while consistent daily rhythms and meal patterns as well as high level of social support have been found as some of the main drivers for succeeding with TRE [10, 11]. However, in-depth knowledge about people’s experiences with engaging with TRE to promote own health during disruptive periods in daily life, e.g. the lockdowns due to COVID-19, is lacking. Interventions such as TRE can be designed as both efficacy and effectiveness studies, often placed on a continuum, with effectiveness studies examining performance in real-life settings [12, 13]. However, no studies have yet examined if and how TRE works in real-life settings during periods of disruptions.. Although TRE is described as a simple eating regimen, there are no qualitative studies to improve our understanding of the lived experiences with TRE as manageable weight loss strategy during disruption to daily routine.

In general, behaviours related to food are often habit-based and flourishing in steady settings [14]. However, with the declaration of COVID-19 as a global pandemic by the World Health Organisation as of March 11th 2020 [15], a growing public health threat and disruptive event interfered with many areas of daily life, resulting in numerous lockdowns worldwide [16]. Lockdowns increased sedentary lifestyles and social distancing [17] which affected daily eating and exercise practices [18–21]. Several studies have examined changes in behaviours related to food during COVID-19, and some have found changes [22, 23] while a number have found a high amount of stability [24–26]. During the Danish COVID-19 lockdowns, more hours were spent on eating and cooking at home, while decreased levels of physical activity and increased levels of boredom and stress led to greater amount of snacking and overeating among some population groups [19]. Particularly, people already having unhealthy eating practices prior to lockdowns [19] and people with overweight have been found to eat unhealthier during lockdowns [27, 28]. This is of concern as excessive energy intake and decreased levels of physical activity can result in weight gain and unfavourable impact on health and well-being [29–31]. As a consequence, negative health effects for a significant number of people alongside the general public health can be expected [32].

The purpose of this qualitative study was to examine if and how the engagement with a TRE regimen among people with overweight who wished to lose weight was affected by Danish COVID-19 lockdowns as an example of disruptions in daily life. Specifically, we wished to examine the robustness of TRE to support weight loss because it is often conceptualised as a manageable and simple regimen to engage with [3] .

## Methods

### Study design

The study was based at Steno Diabetes Center Copenhagen (SDCC) in Denmark and designed as a qualitative sub-study to a randomised controlled trial, the RESET (Restricted Eating Time) study. The trial is registered at Clinicaltrials.gov (identifier: NCT03854656) and details about study design and results can be obtained elsewhere [10, 33]. The study was conducted in accordance to the Declaration of Helsinki and approved by the Ethics Committee of the Capital Region of Denmark (H-18059188). Written informed consent was obtained from each participant before interviews.

### Participants, intervention design and recruitment

The present study included fifteen people enrolled in a three-month TRE intervention at SDCC before the COVID-19 lockdowns. In total, nineteen participants were enrolled in the TRE intervention during lockdowns and invited to participate in this study, but four of them did not respond to our interview invitation. Inclusion criteria included participants being 30 to 70 years of age and either having 1) overweight (BMI ≥25 kg/m^2^) and concurrent prediabetes (HbA_1c_ 39–47 mmol/mol) or 2) obesity (BMI ≥30 kg/m^2^), and a habitual eating/drinking window ≥12 hours (including foods/snacks and energy containing beverages) and an eating/drinking window of ≥14 hours minimum 1 day per week. A detailed list of inclusion and exclusion criteria can be obtained elsewhere [33]. Our aim is to recruit participants at a high risk of type 2 diabetes. Overweight and glycaemia are two partly independent risk factors for type 2 diabetes. Moderate overweight without any indication of dysglycaemia is only associated with a very modest risk of diabetes [34]. and we therefore included prediabetes as an inclusion criterion for individuals with overweight (BMI 25–30 kg/m^2^) in order to ensure that participants are at a risk that is substantial enough to warrant a targeted intervention. Prediabetes is only present in some of the individuals with obesity (BMI ≥30 kg/m^2^), but we also included individuals with obesity without concomitant prediabetes since these individuals are considered at high risk of type 2 diabetes, and weight loss is recommended in this population.

After the test day at baseline, a 7-day free-living assessment period including e.g., measurements of physical activity and glycaemic variability was scheduled. When participants left the research facilities at the baseline test day, they received a bag with a combination lock which contained information about group allocation. On day 7, when all baseline assessments were completed, the participants were called by phone by an investigator and provided with the code for the lock and received instructions about the group allocation. Participants who were allocated to the TRE group were instructed to consume all foods and beverages except water within a self-selected time window of 10 hours per day between 6 am and 8 pm. Furthermore, participants were asked to keep the eating window stable during the week and instructed to select an eating window starting ≥2 hours after habitual wake-up time and end 3 hours before habitual bedtime. Participants in both groups were asked to register start time for the first and time for termination of the last eating/drinking episode (except water) every day. Every week, participants received an e-mail with a link to an online form and asked to register the time for eating/drinking episodes for the previous week. In case participants in the control group restricted the eating window to less than their habitual ≥12 hours/day or if the eating window of participants in the TRE group deviated from their self-selected 10-hour eating window on ≥4 days during the first week, the participant were contacted per telephone to ensure that the participant understood the group allocation. During the first week after group allocation, participants in the TRE group could change their eating window once if they were not satisfied and after the first week no changes were allowed. To ensure the same degree of contact with participants in both groups, no other feedback was provided during the intervention [33] To account for variability in daily eating windows, participants in the TRE group were considered adherent if their eating window was <11 hours/day. Adherence to the intervention is defined as the number/percentage of days the participants’ eating window is < 11 hours/day. *Per protocol* is defined as ≥80% compliance. Compliance data are currently unpublished. The week before subsequent test days, participants received a text message as a reminder. Dietary intake was ad libitum and all participants received and were advised to follow the Danish dietary recommendations [35] but received no other instructions regarding food quantity or quality. All fifteen participants were enrolled in the TRE intervention group and instructed to restrict eating and drinking to the same daily ten-hour window (between 6 AM and 8 PM) but no restrictions regarding food quality or quantity were introduced. However, participants were encouraged to follow the Danish Dietary Recommendations [35]. These include eating plant-rich, varied and not too much as well as including wholegrain foods, less meat, less sweet, salty and fatty foods, choosing legumes and fish, vegetables oils and low-fat dairy products, and drinking water [35]. In the remaining 15 h, participants were only recommended to drink water while no recommendations or restrictions were giving in terms of exercising. Participants did not either receive any support during the intervention period and no modifications were made to support implementation of TRE during lockdowns. Participants were recruited via response to advertisements on digital media or in newspapers and were therefore expected to be considered highly motivated for participation.

### Data collection

A trained researcher in qualitative research interviewed participants using a semi-structured interview guide after the end of the intervention period. The guide included topics on implications of COVID-19 disruptions on TRE engagement and disruptions in daily life such as changes in 1) daily rhythms, 2) eating practices, 3) exercise practices and 4) social life (see Table 1 for overall interview questions). In Denmark, the government ordered two national lockdowns in 2020 to prevent virus transmission: the first on March 11th and the second on December 7th, 2020. During the two similar lockdowns, only essential shops were permitted to remain open. Schools as well as museums, cultural establishments, restaurants, and bars were closed. Employees with a non-critical function in the public sector were sent home to work. Private gatherings were limited to five or ten people, depending on the period. Participants were either interviewed between April and June 2020 during the first Danish lockdown (*n* = 8) or in February 2021 during the second Danish lockdown (*n* = 7) while they were engaged in the intervention. Six interviews were conducted face-to-face with safe social distance, while nine interviews were conducted by phone. All interviews were conducted by the first author and lasted between 25 and 45 min. Interviews were audio recorded and transcribed verbatim.

**Table 1:** Overall interview questions

1. Can you tell me about how you felt to be enrolled in the RESET study during the COVID-19 lockdown? 2. How have you felt about restricting eating and drinking to the same ten-hour window during the COVID-19 lockdown? - When did you place the ten-hour window during the day? - How has it been to eat and drink within the same ten-hour window each day? - Have you been able to maintain it? - When has it been easy? / When has it been challenging? 3. Has the COVID-19 lockdown affected your daily life? If so - how in terms of: - daily life structure? - Work life? - Eating practices? - Exercise practices? - Social life? - Other aspects? 4. What is your perception of time-restricted eating now that you have tried it? 5. Do you have any other comments?	

### Data analysis

The data analysis was inspired by a reflexive thematic analysis approach [36]. First, all transcripts were read carefully by two researchers to obtain familiarity with data. Meaningful text units consisting of participants’ spoken words from each interview were then labelled with emergent codes. During this process, a scheme was developed to organise the emergent codes into initial themes (see Table 2 for example). Initial themes were continuously reviewed for coherence and revised and reviewed by two independent researchers. A high level of agreement was obtained, and only small variances occurred in the interpretation which were clarified by discussion. In the last step, key themes were determined by an iterative process to select the most prominent themes arising from data. The analysis was performed recursively and recontextualization was used to ensure trustworthiness of the study [37] and that themes emerged from the spoken words of participants.

**Table 2:** Example of a scheme to organise initial codes into themes

Themes	Codes	Quotes
Increased level of food consumption	Eaten more Comfort eating Baking more white bread and cake	*’We have been eaten more. I have been baking more. Just like everybody else, we have enjoyed ourselves more with sweets. When we have not been able to do anything, at least we have been able to bake some bread or a cake.’* (Woman, 64 years)
Decreased levels of exercising	Not working out Unable to convert prior exercising routines Spoiled exercise routines due to closed gyms	*’I have not been working out. The lockdown was such a bad timing as I had just developed a nice routine by going to the gym. And I was so close to signing up to spinning classes, but then Denmark was in lockdown. It was so frustrating. When I have decided to exercise in the gym, I am totally fixed on that idea. So, I have not gone for bike rides outside. I have not been exercising at all.’* (Man, 53 years)

## Results

The fifteen participants comprised eight women and seven men, all ethnic Danes and speaking fluent Danish. Participants were 45–68 years of age (mean age: 56.6 years) and characterised by being either retired (*n* = 2), in work (*n* = 9) or unemployed (*n* = 4). None of the unemployed participants had lost their job due to COVID-19 and none of the participants had children living at home. Three key themes arose during the analysis in regard to changes in participants’ engagement in the TRE intervention and the robustness of it: I) Disruptions of COVID-19 lockdown on daily routines and social life, II) Changes in eating practices during COVID-19 lockdowns and III) Changes in exercise practices during COVID-19 lockdowns.

### Disruptions of COVID-19 lockdowns on daily routines and social life

Many (*n* = 9) participants were able to maintain their daily TRE window during lockdown. Some participants expressed how the lockdown had very limited implications on their engagement in TRE as they to a large extent could maintain usual daily routines and rhythms. For those being retired or unemployed this included staying at home as usual while for others it meant going to work:

*I have not been affected by it [COVID-19 lockdown]. I have still been able to go [physically] to work. That, I believe, is a big part of me being able to stick to time-restricted eating. That I have been active during the day. (…*) *So, I have been able to maintain my daily routines.* (Woman, 45 years)

Others (*n* = 6) experienced lockdown disruptions on their TRE engagement since their daily routines changed, primarily because they were forced to work from home. Changes in daily routines often lead to more blurred lines between time for work and private life and less fixed time slots for eating. This challenged some participants’ TRE engagement as TRE often required consistency in daily rhythms and routines. However, lockdowns interrupted this for some participants. A participant reported that his daily routines were turned upside down when he was sent home from work and it interfered negatively with his sleep:

*‘My routines have been reshuffled all the time. Prior to [the COVID-19 lockdown] I went to bed at 10 PM, now it is 11.30 PM and I cannot fall asleep.’* (Man, 53 years)

This participant furthermore expressed how his engagement in TRE decreased due to these changes. However, the participant stressed that despite these changes he managed to keep his eating window*.* This was something most participants were able to do most days, although their daily routines were disrupted. Participants often obtained more spare time during lockdowns and social life was affected by the requirement of social distance. This was constraining to private life, but it facilitated greater engagement to the eating window:

*Time-restricted eating is challenging if you have a social life. If you are going out or visiting people. Then it has been challenging to say at 8 PM ‘now I must stop eating’. But there has not been a lot of this [during COVID-19 lockdown].* (Man 68 years)

Hereby, lockdowns often made it easier to engage in TRE as social barriers such as going out or social dining in late evening contexts were limited.

### Changes in eating practices during COVID-19 lockdowns

Participants who did not experience lockdown disruptions on daily routines did not either report noteworthy changes in eating habits during their TRE window. Participants (*n* = 7) who experienced implications, reported that they had started to eat healthier as they had more time to prepare healthy food, but most participants reported unhealthier eating practices during the window:

*There have not been so many carrots* (while working from the home). *It has been more bread and cake. Some of the new habits, the good ones, have unfortunately slipped in the background because things change when you are sent home from work. When you go to work, you pack your food and take it with you.* (Woman, 64 years)

Increases in snacking during the window was frequently reported. This varied in terms of quality and quantity; from a few nuts to large amounts of white bread and/or cake. During the first lockdown, participants particularly expressed a greater feeling of the need to ‘treat’ themselves with unhealthy snacks. A few participants (*n* = 3) described how the first lockdown made them feel isolated and unhappy which reinforced unhealthy eating in order to relieve these feelings:

*There has been a little devil sitting on my shoulder saying: ‘Yes, this is the right thing to do. You should just eat it. It will solve this crisis’.* (Woman, 55 years)

Such reports were less frequent for participants (*n* = 2) enrolled during the second lockdown. However, across both lockdowns, many participants explained that they consumed more take-away food:

*If we do a count of how many times we have eaten out before COVID-19, and how much we have eaten take-away at home now, then there has been a notable increase in the amount of take-away. It has been increasing steadily. It has been some of the bright spots in daily life during the lockdown.* (Man, 52 years)

Different reasons for a greater intake of take-away were found as some explained that they wanted to support local restaurants and food outlets, while most used it as a treat in daily life because they missed ordinary activities. Together, changes in eating practices due to COVID-19 lockdowns did not interfere much with participants’ adherence to their 10-hour window, but it affected the quality and quantity of food and beverages consumed during the window. This challenged participants’ TRE engagement as they were not able to stick to their ordinary diet.

### Changes in exercise practices during COVID-19 lockdowns

All participants except one exercised less during lockdown. This happened in terms of decreases in daily activities such as cycling to work or walking around at the workplace, but also in terms of decreases in leisure time activities such as swimming, spinning or working out in gyms as these facilities were closed. Notably, for some participants the closing of facilities did not lead to an up-take of other forms of physical activity:

*When I have decided to exercise in the gym, I am totally fixed on that idea. So, I have not gone for bike rides outside. I have not been exercising at all.* (Man, 53 years)

This rigidity meant that a few participants did not exercise during lockdown. Most participants described how they tried to find alternatives that were COVID-19 friendly such as bike rides outside or going for walks:

*Now, I walk maybe 1–3 times per week. So, it is not that I do not do anything at all, but you still have these days when you do not move at all. And then you must pick yourself up and say you must go for a walk. I am bad at that. Therefore, I like to work out in the gym.* (Woman, 51 years)

In general, participants did not exercise to the same extent as prior to lockdown although some exercised at home or outdoor instead. Hereby, participants were not able to convert their pre-lockdown training routines into other types of exercise and keep the same activity level. This challenged participants’ engagement in TRE as it often made them feel less motivated for keeping a healthy lifestyle, which often turned into a vicious circle, increasing unhealthy eating practices too.

## Discussion

In this qualitative study, we showed how a TRE regimen that may appear manageable in even difficult situations became challenging in terms of obtaining a weight loss during Danish COVID-19 lockdowns. Lockdown as an example of disruptions in daily life rapidly affected daily routines for most participants as many were sent home from work and unable to perform usual activities such as socialising, eating out or exercising in gyms. However, most participants were able to manage their eating window but began to eat unhealthier during their window and all began to exercise less. This questions whether TRE with an exclusive focus on the eating window is a recommendable regimen for weight loss during periods of disruption in daily life.

### Time-restricted eating in periods of disruptions in daily life

TRE has been proposed as an eating regimen that may be more sustainable during periods of disruptions in daily life compared to traditional dieting as it encourages the usual food intake and preferences within a shortened time interval without any other restrictions for people to take into account [1]. TRE is often highlighted as an innovative and simple regimen [1, 4]. However, one could question whether TRE is recommendable during periods of disruptions as we found the engagement with health behaviours was highly challenged during COVID-19 lockdowns among even highly motivated people. This was found during daytime with changes in daily routines resulting in eating becoming unhealthier during the window alongside decreases in exercise levels. This is in line with other studies finding increases in unhealthy eating to be higher during COVID-19 lockdowns among individuals with obesity [28] alongside more sedentary behaviour during home confinement [17, 21]. Additionally, home confinement can lead to more frequent snacking which has been associated with both higher intake of calories and higher risk of obesity [38]. However, all participants expressed that exercise practices intrusively changed during lockdown. This changed their TRE engagement and may reflect that exercising and healthy eating often are interconnected.

COVID-19 lockdowns also decreased the level of social evening dining and going out which, on the other hand, eased participants’ engagement with their TRE window during evenings. Social dining in the evening and going out have previously been identified as barriers for TRE performance in daily life [6, 10] which emphasises that TRE may be easier to perform during specific periods of disruptions. This has also been found by others as socialising less during lockdowns have made people meet less with friends and engaging in social eating [20]. Although it was easier for participants to maintain TRE engagement during evenings, lockdowns challenged the general health behaviour engagement as participants changed the food quantity and quality during their eating window. This may have minimised the effects of TRE due to higher calorie consumption. The fact that participants exercised less may also have resulted in a potential lower energy expenditure. Together, this challenges the robustness of the TRE regimen as a simple strategy to implement during periods of disruptions to promote healthier practices.

### Can TRE become successful in terms of weight loss during disruptions in daily life?

Could TRE be modified in ways which would support weight loss also during periods of disruptions? In general, but particularly during disruptions in daily life, it is vital that regimens are robust and consider that healthy behaviour is not solely an individual matter as it is beyond individual capability. In this study, participants did not receive any support during the intervention period and no modifications were made to support healthy behaviours during lockdowns. Our findings revealed that, for whatever cause, more mindful considerations on how to best support people when taking part in a lifestyle intervention are needed, particularly when changes occur in daily life. Therefore, the TRE regimen could benefit of a support component with focus on health behaviours within the eating interval. No single solution will work for all, but our results suggest that the support provided should include more guidance on how to structure daily routines when daily rhythms change. This could make TRE a more robust regimen to obtain healthier practices during disruptive events. Social support could include how to structure safe out-door activities and inspiring guidance about how to structure and make healthy cooking at home suited to the needs of the individual or family instead of using take-away options. Virtual opportunities supporting healthy activities have never been greater which permit initiatives such as online exercising and online cooking classes but also initiatives such as online social support groups or continuous professional support and guidance [29]. Particularly, peer, group and online support have been found helpful during periods of disruptions to develop sustainable healthy behaviour changes and increase their level of resilience [22]. However, it is important that the social support and guidance provided should involve more than an explicit focus on diet and exercising as many participants described that the missing structure in daily life was the most essential factor for decreased engagement. It is important that the guidance and support is offered at a local level, which could include work organisations as well as educational and school settings because these are some of the daily settings people spend most of their time in. Additionally, it is vital to differentiate the support and guidance to the needs and resources of different target groups to minimise social inequalities as people have different needs for support when it comes to daily life practices, making healthy choices and engaging in healthy behaviour. Some may need structured support, while others may need support more occasionally. To differentiate the support and to guide people with overweight are of particular importance as weight gain can lead to higher risk of developing non-communicable diseases and worsening of already existing ones [39].

Additionally, knowledge from previous epidemic outbreaks has revealed that as epidemics progress, a vital need emerges to refocus public health recommendations and activities to include more than the management of clinical issues by a specific focus on health behaviours [30, 40]. The relevance of this is confirmed in our study, but during lockdowns, worldwide, minimal public focus, if any, was pointed towards broader issues of health behaviour. Disruptions is not only great interfering pandemics with lockdowns but could also be individual disruptions such as stressful situations, severe illness and divorce or other serious life changing events. However, we do not know if this would lead to similar results, but it is worth mentioning as transferable situations for disruptions. Therefore, the concept of TRE could benefit of focusing beyond the ‘time’ as in modern, changeable daily lives disruptions are often present. It is essential that disruptions are considered in future health promotion interventions by taking the individual life situation into account when introducing strategies such as TRE. This is important to not only make health interventions successful during periods of disruptions, but also to make them work in ordinary daily life structure which is often characterised by change and shifting levels of stability. Maybe even more participant would have continued to be successful with TRE despite disruptions if potential deviations to daily routine were discussed when TRE was initially introduced. This could for example include participants’ being provided TRE instructions, and in addition to this being asked to talk about barriers that could get in the way of their TRE window (e.g., a change in work schedule, sleep disruptions, illness, getting fired). Participants could then proactively identify how to maintain TRE successfully. This could be both individually with a health care professional or in e.g., peer support groups to better address how TRE can be adapted to changes in daily life.

### Strength and limitations

This study has several strengths as well as limitations. Regarding strengths, to the best our knowledge, this study is the first to examine in-depth understanding of people’s experiences with engaging with TRE during periods of disruptions to determine the robustness of the regimen. This is of great value for the usefulness and sustainability of TRE in real life settings. Moreover, the richness of our qualitative data added nuances to the field and ensured specific recommendations for how TRE can be designed more robust to increase public health areas by people obtaining healthier practices and general lifestyles. However, our study also has several limitations that deserve further attention. Many participants were above 55 years of age and none of them had children living at home. Our sample is hereby not representative for especially younger age groups. Another limitation of this study was how participants were recruited via advertisement and hereby characterised by a high level of motivation for participation. This stresses how the implications found in this study could be even more present among people enrolled as a part of treatment which highlight the crucial need of addressing the identified implications in the design of future studies.

## Conclusions

Examining TRE during the Danish COVID-19 lockdowns as an example of disruptions in daily life has provided some markers for the robustness of TRE in real-life settings. Although the TRE time window was manageable for most, TRE as a weight loss strategy was highly challenged by ‘disruption-related’ unhealthy behaviours during the eating interval. Differentiated support and adjustments of the TRE regimen are needed for successful uptake and/or maintenance of healthy behaviours. Thereby the TRE regimen could be supplemented with a focus on healthy behaviours to ensure higher levels of robustness in times of disruptions.

## Data Availability

The datasets generated and analysed during the current study are not publicly available due to participants being guaranteed confidentiality, but data are available from the corresponding author on reasonable request.

## Abbreviations

TRE: Time-restricted eating
RCT: Randomised controlled trial

## References

[1] Ealey KN, Phillips J, Sung H-K. COVID-19 and obesity: Fighting two pandemics with intermittent fasting. Trends Endocrinol Metab. 2021;32(9):706–20. 10.1016/j.tem.2021.06.004 PMC8226104 34275726

[2] Parr EB, Devlin BL, Radford BE, Hawley JA (2020). A delayed morning and earlier evening time-restricted feeding protocol for improving glycemic control and dietary adherence in men with overweight/obesity: a randomized controlled trial. Nutrients.

[3] Hawley JA, Sassone-Corsi P, Zierath JR. Chrono-nutrition for the prevention and treatment of obesity and type 2 diabetes: from mice to men. Diabetologia. 2020;63(11):2253–59 10.1007/s00125-020-05238-w 32761356

[4] Moon S, Kang J, Kim SH, Chung HS, Kim YJ, Yu JM, Cho ST, Oh C-M, Kim T (2020). Beneficial effects of time-restricted eating on metabolic diseases: a systemic review and meta-analysis. Nutrients.

[5] Heilbronn LK, Regmi P. Will delaying breakfast mitigate the metabolic health benefits of time-restricted eating? Obesity. 2020. 10.1002/oby.22776 32438489

[6] Regmi P, Heilbronn LK. Time Restricted eating: Benefits, Mechanisms, and Challenges in Translation. Iscience 2020;23(6):101161. 10.1016/j.isci.2020.101161 PMC7262456 32480126

[7] Longo VD, Panda S (2016). Fasting, circadian rhythms, and time-restricted feeding in healthy lifespan. Cell Metab.

[8] Cienfuegos S, McStay M, Gabel K, Varady KA (2022). Time restricted eating for the prevention of type 2 diabetes. J Physiol.

[9] Manoogian ENC, Chow LS, Taub PR, Laferrère B, Panda S (2022). Time-restricted eating for the prevention and management of metabolic diseases. Endocr Rev.

[10] Bjerre N, Holm L, Quist JS, Færch K, Hempler NF. Watching, keeping and squeezing time to lose weight: implications of time-restricted eating in daily life. Appetite. 2021;105138. 10.1016/j.appet.2021.105138 33524440

[11] O'Connor SG, Boyd P, Bailey CP, Shams-White MM, Agurs-Collins T, Hall K, et al. Perspective: time-restricted eating compared with caloric restriction: potential facilitators and barriers of long-term weight loss maintenance. Adv Nutr. 2021. 10.1093/advances/nmaa168 PMC8009736 33463673

[12] Revicki DA, Frank L (1999). Pharmacoeconomic evaluation in the real world. Pharmacoeconomics.

[13] Singal AG, Higgins PD, Waljee AK (2014). A primer on effectiveness and efficacy trials. Clin Transl Gastroenterol.

[14] Verplanken B, Aarts H (1999). Habit, attitude, and planned behaviour: is habit an empty construct or an interesting case of goal-directed automaticity?. Eur Rev Soc Psychol.

[15] Cucinotta D, Vanelli M (2020). WHO declares COVID-19 a pandemic. Acta bio-medica: Atenei Parmensis.

[16] He Z (2020). What further should be done to control COVID-19 outbreaks in addition to cases isolation and contact tracing measures?. BMC Med.

[17] Stockwell S, Trott M, Tully M, Shin J, Barnett Y, Butler L, McDermott D, Schuch F, Smith L (2021). Changes in physical activity and sedentary behaviours from before to during the COVID-19 pandemic lockdown: a systematic review. BMJ Open Sport Exerc Med.

[18] Ammar A, Brach M, Trabelsi K, Chtourou H, Boukhris O, Masmoudi L, Bouaziz B, Bentlage E, How D, Ahmed M (2020). Effects of COVID-19 home confinement on eating behaviour and physical activity: results of the ECLB-COVID19 international online survey. Nutrients.

[19] Giacalone D, Frøst MB, Rodríguez-Pérez C (2020). Reported changes in dietary habits during the Covid-19 lockdown in the Danish population: the Danish COVIDiet study. Front Nutr.

[20] Dor-Haim H, Katzburg S, Revach P, Levin H, Barak S (2021). The impact of COVID-19 lockdown on physical activity and weight gain among active adult population in Israel. A Cross-Sectional Study.

[21] Karageorghis CI, Bird JM, Hutchinson JC, Hamer M, Delevoye-Turrell YN, Guérin SM, Mullin EM, Mellano KT, Parsons-Smith RL, Terry VR (2021). Physical activity and mental well-being under COVID-19 lockdown: a cross-sectional multination study. BMC Public Health.

[22] Avery A, Toon J, Kent J, Holloway L, Lavin J, Bennett S-E (2021). Impact of COVID-19 on health-related behaviours, well-being and weight management. BMC Public Health.

[23] Van der Werf ET, Busch M, Jong MC, Hoenders H (2021). Lifestyle changes during the first wave of the COVID-19 pandemic: a cross-sectional survey in the Netherlands. BMC Public Health.

[24] Poelman MP, Gillebaart M, Schlinkert C, Dijkstra SC, Derksen E, Mensink F, Hermans RC, Aardening P, de Ridder D, de Vet E (2021). Eating behavior and food purchases during the COVID-19 lockdown: a cross-sectional study among adults in the Netherlands. Appetite.

[25] Janssen M, Chang BP, Hristov H, Pravst I, Profeta A, Millard J (2021). Changes in food consumption during the COVID-19 pandemic: analysis of consumer survey data from the first lockdown period in Denmark, Germany, and Slovenia. Front Nutr.

[26] Chenarides L, Grebitus C, Lusk JL, Printezis I (2021). Food consumption behavior during the COVID-19 pandemic. Agribusiness.

[27] Poelman MP, Gillebaart M, Schlinkert C, Dijkstra SC, Derksen E, Mensink F, et al. Eating behavior and food purchases during the COVID-19 lockdown: a cross-sectional study among adults in the Netherlands. Appetite. 2020;105002. 10.1016/j.appet.2020.105002 PMC7554484 33068668

[28] Ashby NJ (2020). Impact of the COVID-19 pandemic on unhealthy eating in populations with obesity. Obesity.

[29] Bhutani S, Cooper JA. COVID-19 related home confinement in adults: weight gain risks and opportunities. Obesity. 2020. 10.1002/oby.22904 PMC7276847 32428295

[30] Naja F, Hamadeh R. Nutrition amid the COVID-19 pandemic: a multi-level framework for action. Eur J Clin Nutr. 2020:1–5. 10.1038/s41430-020-0634-3 PMC7167535 32313188

[31] Yanovski JA, Yanovski SZ, Sovik KN, Nguyen TT, O'Neil PM, Sebring NG (2000). A prospective study of holiday weight gain. N Engl J Med.

[32] Lytras T, Tsiodras S (2021). Lockdowns and the COVID-19 pandemic: what is the endgame?. Scand J Public Health.

[33] Quist JS, Jensen MM, Clemmensen KKB, Pedersen H, Bjerre N, Størling J, Blond MB, Wewer Albrechtsen NJ, Holst JJ, Torekov SS (2020). Protocol for a single-Centre, parallel-group, randomised, controlled, superiority trial on the effects of time-restricted eating on body weight, behaviour and metabolism in individuals at high risk of type 2 diabetes: the REStricted eating time (RESET) study. BMJ Open.

[34] Schmidt MI, Bracco PA, Yudkin JS, Bensenor IM, Griep RH, Barreto SM (2019). Intermediate hyperglycaemia to predict progression to type 2 diabetes (ELSA-Brasil): an occupational cohort study in Brazil. Lancet Diabetes Endocrinol.

[35] The Official Dietary Guidelines. https://altomkost.dk/english/#c41067.

[36] Braun V, Clarke V (2019). Reflecting on reflexive thematic analysis. Qual Res Sport Exerc Health.

[37] Nowell LS, Norris JM, White DE, Moules NJ (2017). Thematic analysis: striving to meet the trustworthiness criteria. Int J Qual Methods.

[38] Scully M, Dixon H, Wakefield M (2009). Association between commercial television exposure and fast-food consumption among adults. Public Health Nutr.

[39] Clemmensen C, Petersen MB, Sørensen TI (2020). Will the COVID-19 pandemic worsen the obesity epidemic?. Nat Rev Endocrinol.

[40] Gamage SD, Kralovic SM, Roselle GA (2009). Emerging infectious diseases: concepts in preparing for and responding to the next microbial threat.

